# The role of TGF-β superfamily in endometriosis: a systematic review

**DOI:** 10.3389/fimmu.2025.1638604

**Published:** 2025-08-12

**Authors:** Xinyi Xu, Jun Li, He Lin, Zhe Lin, Guangcheng Ji

**Affiliations:** ^1^ College of Pharmacy, Changchun University of Chinese Medicine, Changchun, China; ^2^ School of Rehabilitation Medicine, Changchun University of Chinese Medicine, Changchun, China

**Keywords:** TGF-β superfamily, endometriosis, signal transduction, gene expression, epigenetics

## Abstract

**Introduction:**

Endometriosis is a prevalent chronic gynecological disorder. Globally, endometriosis affects approximately 5–10% of women of reproductive age, leading to symptoms such as dysmenorrhea, chronic pelvic pain, and infertility. While the precise etiology of endometriosis remains unclear, various etiological theories have been suggested to explain the condition’s development. Recent research has focused on the TGF-β superfamily, which regulates cell proliferation, differentiation, migration, and immune modulation, and is increasingly recognized as a key contributor to the pathogenesis of endometriosis.

**Methods:**

This review provides a comprehensive examination of TGF-β superfamily in endometriotic lesions. According to the recommendations of the Preferred Reporting Project for Systematic Review and Meta-Analysis (PRISMA) guidelines, a literature search was conducted in the PubMed and Web of Science database until April 30, 2025.

**Results:**

TGF-β superfamily contributes not only to the adhesion, invasion, and proliferation of ectopic endometrial cells but also to the mediation of fibrosis, immune modulation, and angiogenesis within endometriotic lesions. Considering the parallels between endometriosis and malignant processes, including local invasion and abnormal tissue growth, analyzing the TGF-β-mediated mechanisms offers new insights into disease progression and its oncological parallels. Exploration of TGF-β-dependent biomarkers and targeted inhibitors holds potential in advancing more effective diagnostic and therapeutic approaches.

**Discussion:**

This study emphasizes further research into TGF-β and related pathways, potentially paving the way for innovative, targeted therapeutic strategies aimed at managing endometriosis, reducing recurrence rates, and enhancing the quality of life for affected women.

## 1 Introduction

Endometriosis (EMS) is a common chronic gynecological condition. Its hallmark is the presence of functional endometrial tissue outside the uterine cavity. This tissue is primarily located in the ovaries, peritoneum, uterosacral ligaments, rectovaginal pouch, and other areas of the body (1). The three main forms of endometriosis are superficial peritoneal endometriosis (SPE), deep infiltrating endometriosis (DIE), and ovarian endometriosis (OE) (2). In addition to causing increased localized inflammation and estrogen production, EMS affects 5–10% of women globally of childbearing age, leading to a variety of pains such as menstrual cramps, dysuria, dyschezia, and abdominal pain (3). Inflammation, cysts, scar tissue, and adhesions may lead to complications such as intestinal issues, chronic pelvic inflammatory disease, or infertility (4). Currently, Progestins, oral contraceptives, and gonadotropin-releasing hormone agonists are used to manipulate hormones to suppress menstruation and ovulation, thereby suppressing lesions. Alternatively, surgical removal of deep nodules, ovarian cysts, and peritoneal implant is employed to alleviate pain (5). However, approximately 75% of women experience a recurrence of related symptoms within two years after undergoing surgery, and medications currently used to treat EMS often have adverse side effects (6). Moreover, EMS shares biological characteristics with cancer, including dissemination, invasion, and proliferation, although clinically and pathologically presenting as benign. Recent studies have shown that progressive endometriosis may lead to endometriosis-associated ovarian cancer (EAOC), including encompassing ovarian clear cell carcinoma (OCCC) and ovarian endometrioid carcinoma (OEC) (6). The pathophysiology of EMS remains elusive, while attention to the condition has increased recently.

Several studies have demonstrated differential cytokine expression in women with endometriosis, observed in serum, peritoneal fluid, and ectopic lesions. These cytokines promote endometriotic cell survival, growth, invasion, differentiation, angiogenesis, and other processes implicated in the onset and progression of endometriosis (7). The transforming growth factor β (TGF-β) superfamily is one of the cytokine families involved in endometriosis. It has been reported that TGF-β1 induced an increase in prolactin levels and significant decidual-like changes in ectopic endometrial stromal cells (ESCs). Women with mild endometriosis exhibited downregulation of progesterone receptor expression in the endometrium due to elevated levels of TGF-β1 (8). Other studies have shown that the peritoneal fluid of endometriosis cases exhibited significantly elevated levels of soluble endothelial protein, growth differentiation factor 15 (GDF-15), and TGF-β1 compared to the control group. Additionally, patients with advanced endometriosis showed significantly higher serum levels of GDF-15 compared to those in the early-stage group, suggesting a potential role for TGF-β-dependent signaling, and serum GDF-15 could serve as a potential biomarker for assessing endometriosis severity (9).

To address this pervasive but still elusive pathogenesis, current research into endometriosis necessitates a comprehensive understanding of the role of cytokines in the disease. A survey of the existing literature indicates that while research on this topic has been ongoing since 1994, its scope and depth remain limited. In response to the ongoing exploration by the scientific community, it is necessary to more fully integrate TGF-β superfamily role in endometriosis, and update to the existing literature review. Therefore, this review covers both clinical findings and experimental data, offering a comprehensive summary of recent studies on the TGF-β superfamily linked to endometriosis. It is beneficial to shed light on the function of the TGF-β superfamily in the pathogenesis of endometriosis and offer fresh ideas for possible treatment approaches.

## 2 Methods

The objective of this systematic review is to investigate the TGF-β superfamily, with a particular focus on how TGF-β, activins, statins, and bone morphogenetic proteins (BMPs) contribute to the development and maintenance of endometriosis lesions. According to the recommendations of the Preferred Reporting Project for Systematic Review and Meta-Analysis (PRISMA) guidelines (10), a literature search was conducted in the PubMed and Web of Science database to systematically review the primary research articles published up to April 30, 2025 using the following search terms: “endometriosis” AND “TGF-β” or “endometriosis” AND “BMP” or “endometriosis” AND “activin” or “endometriosis” AND “inhibin” or “endometriosis” AND “GDF-8” or “endometriosis” AND “nodal” or “endometriosis” AND “AMH”.

All authors jointly formulated and agreed on the inclusion and exclusion criteria. Studies that met the following criteria included: (a) English articles; (b) Original full-text articles; (c) To focus on the role of TGF-β superfamily in EMS. In addition, the citations of the identified studies were reviewed to incorporate more relevant articles. The exclusion criteria included: (a) Non-English articles; (b) Non-original research articles; (c) Withdrawn publications; (d) No full manuscripts available; (e) No direct relationship with TGF-β or EMS. Finally, a total of 61 articles were included in the review (**Supplementary Figure S1**, **Supplementary Table S1**).

## 3 TGF-β superfamily-mediated signaling

### 3.1 Ligands of TGF-β superfamily

#### 3.1.1 TGF-β subfamily

TGF-βs, activin/inhibin, Node growth and differentiation factors (Nodal), the muscle growth inhibitor (GDF-8), and a few GDF members are all part of the TGF-β subfamily (**Table 1**) (22). Three TGF-β isoforms (TGF-β1, TGF-β2, and TGF-β3) are found in mammals, while TGF-β4 and TGF-β5 are found in birds and amphibians. Disulfide bonds bind the two structurally identical or similar subunits in each TGF-β family, each subunit having a molecular weight of 12 kDa. The mature ligands of the three TGF-β isoforms exhibit conservation in their amino acid sequences, despite being encoded by three distinct genes (11). Epithelial cells undergo apoptosis when exposed to TGF-β, a strong growth inhibitor for a variety of cell types. *In vivo*, it induces fibrosis in different tissues by promoting the synthesis of extracellular matrix proteins (12). Activin and inhibin share a common β-subunit. Activin is a heterodimer of two β-subunits covalently linked by a disulfide bond, whereas inhibin is a heterodimer of one α-subunit and one β-subunit (13). Activin stimulates the release of follicle-stimulating hormone (FSH) from the pituitary gland and contributes to the formation of dorsal mesoderm during embryogenesis. In contrast, inhibin exhibits extra-pituitary effects as an antagonist of activin. It is initially discovered as a cytokine that inhibits FSH secretion by the pituitary gland (12). The physiological processes of germ cell development, oocyte maturation, follicular development, ovulation, decidualization, endometrial tolerance, embryo implantation, placentation, and postmenstrual endometrial repair are all influenced by activin and inhibin (23–25). The expression of the multifunctional factor Nodal is seen in tissues with high turnover, such as the endometrium, and is crucial for controlling placental development. Nodal primarily promotes the development of the dorsal mesoderm and establishes left-right asymmetry in the developing embryo (14). According to Halban’s theory of benign metastasis, endometriotic cells may be present in lymphatic vessels and lymph nodes. This theory suggests a possible mechanism for the spread of endometriosis beyond the pelvic region. Nevertheless, Nodal is expressed in malignant tumors, and alterations in its expression correlate with cancer aggressiveness and progression. There are also case reports of individuals with endometriosis-associated malignant transformation that mimic tumors, presenting with para-aortic lymph node involvement (15, 26). The endometrium of women with EMS exhibits a subtle alteration in the Nodal signaling pathway, which may be useful in distinguishing between malignancy and the highly proliferative endometriotic cells (27). In addition, muscle cells produce GDF-8, also known as myostatin, which is a muscle growth inhibitor and is involved in cell differentiation and proliferation. Studies have notably demonstrated a close relationship between GDF-8 regulation and folliculogenesis, highlighting its role as a key growth factor in this process (16). GDF-8 plays an important role in ovarian steroidogenesis by mediating the granulosa cell response to gonadotropins during the follicular growth phase (17). Female patients with EMS exhibit fewer follicles and varying degrees of oocyte quality impairment compared to healthy individuals, which indicates lower clinical pregnancy rates (28). Further studies are needed to ascertain whether GDF-8 contributes to this impairment. The mRNA encoding the muscle growth inhibitor was found to be highly expressed in patients with deep invasive endometriosis (29). This finding provides physiological support for the involvement of GDF-8 in the onset and progression of endometriosis. However, this topic will not be extensively covered in this article due to limited research on the relationship between GDF-8 and endometriosis.

**Table 1:** Ligands of TGF-β superfamily and their functions.

Subfamily	Ligand	IsoformsMembers	Molecular Structure	Function	References
TGF-β Subfamily	TGF-βs	TGF-β1, TGF-β2, TGF-β3	Disulfide-bonded homodimer, 12 kDa per subunit	Growth inhibition, apoptosis, fibrosis	(11, 12)
	Activin	Activin A, Activin B	Heterodimer of two β-subunits	FSH release, dorsal mesoderm formation	(12, 13)
	Inhibin	Inhibin A, Inhibin B	Heterodimer of one α-subunit and one β-subunit	Inhibition of FSH secretion, antagonizes Activin	(12, 13)
	Nodal	Nodal	Structurally related to Activin	Left-right asymmetry, placental development	(14, 15)
	GDF-8 (Myostatin)	GDF-8	Homodimer, related to TGF-β	Muscle growth inhibition, folliculogenesis	(16, 17)
	few GDF members	GDF-1, GDF-3, GDF-9, GDF-11			
BMP Subfamily	BMPs	BMP2 and BMP4; BMP5, BMP6, BMP7, BMP8a, and BMP8b; BMP9 and BMP10; BMP12, BMP13, and BMP14	Homodimer/Heterodimer with cysteine knot structure	Ovarian function, bone formation, endometrial remodeling	(18, 19)
	most GDF members	GDF-2,GDF-4, GDF-5, GDF-6, GDF-7,GDF-10,GDF-12,GDF-13,GDF-14			
	AMH	AMH	Dimeric glycoprotein	Indicator of ovarian reserve, ovarian function regulation	(20, 21)
	GDF-15	GDF-15	Distant TGF-β family member	Potential biomarker for EMS	(9)

#### 3.1.2 BMP subfamily

BMP, Anti-Mullerian hormone (AMH), and most GDF members constitute the BMP subfamily (**Table 1**) (22). BMP has the ability to regulate ovarian function, embryogenesis, and bone formation (18). BMPs can be classified into four subgroups based on sequence similarity: BMP2 and BMP4; BMP5, BMP6, BMP7, BMP8a, and BMP8b; BMP9 and BMP10; BMP12, BMP13, and BMP14. Seventh cysteine residue is capable of forming disulfide bonds with other monomers, facilitating the formation of both homodimers and heterodimers. The six cysteine residues within the cysteine junction motif in BMP contribute to disulfide bond formation within the molecule (30). Substantial evidence demonstrates that BMP regulates endometrial remodeling by controlling various molecular targets (19). GDF-1, GDF-3, GDF-8, GDF-9, and GDF-11 belong to the TGF-β superfamily, with the remaining members having structural similarities to BMP and being classified as BMP members. Notably, GDF-15 is classified under the distant TGF-β superfamily, and its mechanism of action does not involve the activation of TGF-β superfamily. Elevated serum levels of GDF-15 were observed in the late stages compared to the early stages, and significantly higher levels were found in the peritoneal fluid of infertile women with severe EMS. Therefore, serum GDF-15 shows promise as a potential biomarker for assessing EMS severity (9). AMH is a dimeric glycoprotein expressed in the granulosa cells of developing follicles in adult females (20). One accurate indicator of ovarian reserve is the serum level of AMH. Research has shown that serum AMH levels in women with OE are significantly lower compared to fertile controls (21). As a result, the level of AMH can be used to determine the optimal surgical technique for treating OE, thereby minimizing the impact on the woman’s ovarian tissue (31).

### 3.2 Receptors

Type I and type II receptors of the TGF-β superfamily facilitate signaling through heterotetrameric complexes formed by two type I and two type II receptors, enabling mature TGF-β superfamily ligands to transmit signals. These bispecific kinase receptors, also known as transmembrane kinases, exhibit structural similarities with both serine/threonine and tyrosine kinases (32). The transmembrane structural domain of the type I receptor is separated from its kinase structural domain by a short Gly-Ser-rich sequence, known as the GS domain in the vicinity of the membrane (33). After ligand binding in the tetrameric receptor complex, the kinase of the type II receptor phosphorylates the GS domain, initiating activation of the type I receptor kinase, which then phosphorylates intracellular substrates (34). As a result, the type I receptor in the TGF-β superfamily signaling pathway functions downstream of the type II receptor, determining the specificity of intracellular signaling induced by cytokines within the TGF-β superfamily (12). ALK-1(ActRL1), ALK-2(ActR-IA), ALK-3(BMPR-IA), ALK-4(ActR-IB), ALK-5(TβR-I), ALK-6(BMPR-IB), and ALK-7(ActR-IC) are the seven type I receptors found in mammals. Additionally, TβR-II, ActR-II, ActR-IIB, BMPR-II, and AMHR-II are the five characterized type II receptors (8). A number of co-receptors also influence signaling, including rejection guidance molecule (RGM), neuropilin-1 (NRP1), β-glycan (TβR-III), Endoglin, BMP and activin membrane-bound inhibitor (BAMBI), the EGF-CFC family protein Cripto, and CD109 (8).

### 3.3 Ligand synthesis and activation

Activation of the latent complex is a crucial biological checkpoint that regulates TGF-β bioavailability. In the basal state, TGF-β superfamily ligands are sequestered in the extracellular matrix (ECM) as latent forms (35). Precursor molecules, composed of a signal peptide, the latency-associated peptide (LAP) pre-structural domain, and mature TGF-β, are synthesized to facilitate the binding of active TGF-β to its receptor. Proteolytic cleavage of basic residues in the precursor leads to the formation of pro-TGF-β after the signal peptide is removed. The pre-structural domains of pro-TGF-β are cleaved from the mature polypeptide, allowing it to dimerize into homo- and heterodimeric proteins. The latent TGF-β complex forms a large latent complex (LLC) with latent TGF-β binding protein (LTBP) via disulfide bonds. Upon binding to the ECM membrane protein GARP, the LLC releases active TGF-β through integrins (8, 34, 36).

### 3.4 Two Smad signals

**Figure 1:**
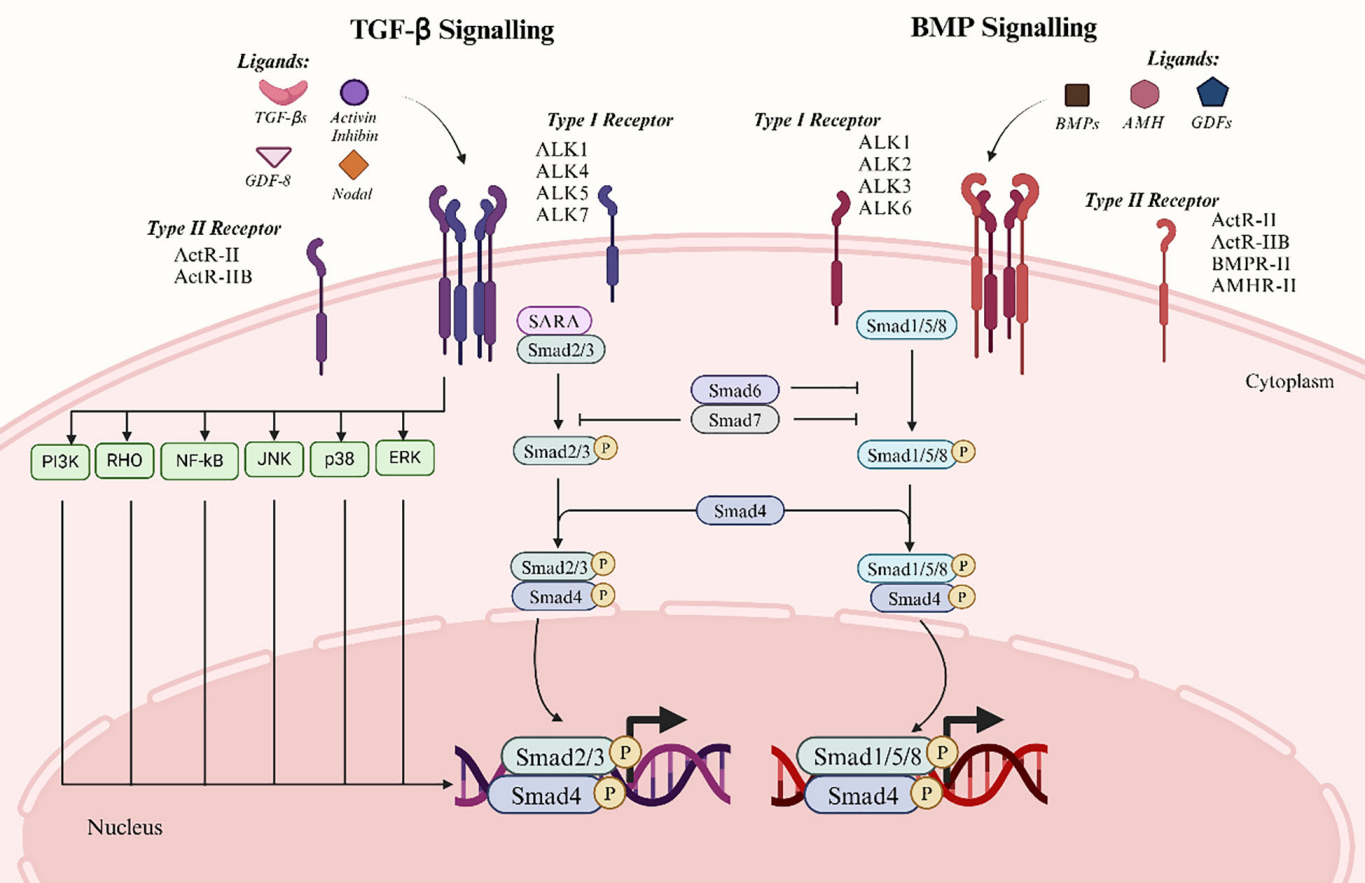
Two major signalling pathways of the TGF-β superfamily. The left panel depicts the TGF-β signaling pathway, which primarily involves ligands such as TGF-β, Activin, Inhibin, and Nodal. The right panel illustrates the BMP signaling pathway, involving ligands including BMPs, AMH, and GDFs. The canonical signaling process begins with ligand binding to type II receptors, which subsequently recruit and activate type I receptors. Activated type I receptors phosphorylate and activate downstream Smad proteins. In the TGF-β signaling pathway, Smad2/3 are phosphorylated and form a complex with Smad4, which translocates to the nucleus to regulate gene expression. Similarly, in the BMP signaling pathway, Smad1/5/8 are phosphorylated and bind Smad4 before entering the nucleus. Smad6 and Smad7 act as inhibitory Smads, attenuating signaling via a negative feedback mechanism. Furthermore, TGF-β signaling can activate non-Smad-dependent pathways, including PI3K, JNK, and ERK, to mediate additional cellular responses.

Major signaling molecules known as Smad proteins act downstream of serine/threonine kinase receptors. Transducing TGF-β signals from cell surface receptors to the nucleus is a critical function of the Smad family proteins (12). Distinct Smads mediate the signaling of specific TGF-β superfamily members. Upon ligand binding to their receptors, Smads translocate into the nucleus and either co-activate or repress transcription of their target genes. Consequently, Smads act as signal transducers for the TGF-β superfamily, modulating gene expression and serving as key effectors of TGF-β signaling (8). Two types of Smad signaling are induced by TGF-β superfamily proteins including classical Smad signaling and Smad-independent signaling (**Figure 1**).

#### 3.4.1 Traditional Smad pathway for signaling

Based on their functions, the eight Smad proteins encoded in mammalian genes mediate signaling by TGF-β superfamily members and can be categorized into three subtypes: inhibitory Smads (I-Smads), common pathway Smads (Co-Smads), and receptor-regulated Smads (R-Smads). Type I receptors activate R-Smads to form transient complexes. R-Smads are further divided into two groups: AR-Smads and BR-Smads. AR-Smads like Smad2 and Smad3 mediate signaling of TGF-β, activin, GDF-8, GDF-11, and Nodal, while BR-Smads such as Smad1, Smad5, and Smad8 mediate BMP signaling. Co-Smad, exemplified by Smad4, is essential across all TGF-β superfamily signaling types, often partnering with R-Smads to regulate gene expression. I-Smads like Smad6 and Smad7, which bind to activated type I receptors, thereby inhibiting or modulating TGF-β superfamily signaling (37).

In classical signaling, R-Smads are phosphorylated by the type I receptor kinase upon receptor activation. This phosphorylation leads to the formation of a heterotrimeric complex consisting of phosphorylated R-Smad and Co-Smad (32). Together with Smad4, this complex translocates to the nucleus, where it interacts with specific cofactors and regulatory proteins to form transcriptional complexes that modulate target gene expression (38). Moreover, Smads activate I-Smads, initiating a negative feedback loop to attenuate signaling. Upon activation, the type I receptor binds to I-Smads to inhibit R-Smad activation. Smad6 competes with R-Smads for receptor binding and prevents Co-Smad complex formation. Specifically, Smad6 predominantly inhibits BMP signaling, while Smad7 suppresses both TGF-β and BMP pathways (12).

#### 3.4.2 Unconventional pathway for Smad signaling

When TGF-β-related ligands bind to their receptors, non-classical signaling pathways are initiated. These pathways, known as Smad-independent signaling, involve downstream effectors such as TRAF4, TRAF6, TAK1, p38 MAPK, RHO, PI3K-AKT, ERK, JNK, and NF-κB. These pathways are implicated indirectly in processes including apoptosis, migration, proliferation, differentiation, and matrix formation (11). The primary connection between the p38 MAPK and JNK signaling pathways and the cellular stress response is their involvement in cell invasion and migration in addition to apoptosis and survival. The invasive potential and lesion expansion in EMS may be encouraged by abnormal activation of these signaling pathways. The proliferation and survival of embryonic stem cells are intimately linked to the PI3K-AKT and ERK pathways. By encouraging cell survival and anti-apoptotic mechanisms, the PI3K-AKT signaling pathway causes excessive cell proliferation and lesion tissue formation in EMS. Conversely, the ERK signaling pathway further encourages the aberrant proliferation of ESCs by regulating cell cycle and proliferation-related gene expression, thereby encouraging cell proliferation and lesion tissue formation in EMS (39). And TGF-β overexpression improves ESCs migration and invasiveness as well as activates the ERK/MAPK signaling pathway in embryonic stem cells. It also shows that the ERK/MAPK signaling pathway mediates the influence of TGF-β on the proliferative, migratory, and invasive abilities of ESCs. Some researchers have discovered that TGF-β overexpression increased the migratory and invasive abilities of ESCs, as well as the activation of the ERK/MAPK signaling pathway in embryonic stem cells (39, 40). NF-κB is a pivotal transcription factor regulating genes involved in inflammation, cell survival, and proliferation. Its activation in endometriosis exacerbates the disease by promoting abnormal cell proliferation and chronic inflammation. Experimental evidence suggested that TGF-β-mediated NF-κB-p65 activation in endometriosis cells was attenuated upon downregulation of TAK1. Moreover, inhibition of TAK1 targeting reduces the inhibitory effect of TGF-β1 non-classical signaling via the NF-κB/Smad7 axis on human endometriosis cell proliferation and promotes autophagic cell death (41).

The Smad-independent pathway represents an alternative mechanism by which the TGF-β signaling pathway influences cellular functions, distinct from the conventional Smad-dependent pathway (40). The interaction of multiple signaling pathways produces intricate and diverse cellular responses to external stimuli. The TGF-β superfamily is involved in the complex pathological processes of EMS through various intricate molecular mechanisms. Investigation into these pathways not only advances our understanding of the condition, but also suggests potential therapeutic targets and intervention strategies, thereby opening critical new avenues for future research and clinical care.

## 4 TGF-β superfamily expression in endometrium

The expression levels of members of the TGF-β superfamily vary across different tissues and cells in endometriosis, depending on the stage of the menstrual cycle (**Table 2**). Studies have consistently demonstrated that elevated TGF-β levels in the serum, glandular cells, peritoneum, and ectopic ESCs of patients with endometriosis. Moreover, activin and inhibin exhibited variable expression during the menstrual cycle and are prominently expressed in peritoneal fluid. And women with endometriosis demonstrated altered BMP-6 expression in their ovarian theca cells (42). As a multifunctional cytokine, Nodal played a key role in the biological process of ovarian endometriosis-cancerous lesions (43).

**Table 2:** Expression of TGF-βs, activin and inhibin, BMPs.

Factor	Expression position	Expression changes	Role in endometriosis	References
TGF-βs	Serum, Peritoneal fluid, Endometrium, Ectopic endometrium, Glandular cells, Macrophages, Stromal cells	TGF-β1 shows a slight increase during the menstrual period and then remains relatively stable. TGF-β2 increases sharply from the early secretory phase to the secretory phase, and again during the menstrual phase. TGF-β3 rises from the secretory phase to the menstrual phase and remains elevated throughout the proliferative phase.	Endothelial physiological regulation, promoting cell proliferation and migration, tissue remodeling, immune regulation	(41–53)
Activin and Inhibin	Peritoneal fluid, Cystic fluid, Endometrial epithelial and stromal cells	Inhibin A and activin A levels increase during the secretory phase. Inhibin α mRNA expression decreases, while activin βA mRNA expression increases in the ectopic endometrium.	Affecting embryo implantation, regulating stromal cell differentiation, and serves as a diagnostic marker.	(29, 54–64)
BMPs	Ectopic endometrial stroma and epithelial cells, Granulosa cells, Follicular cells surrounding oocytes	BMP6 is highly expressed in the ectopic endometrium, while BMP7 is highly expressed during the proliferative and secretory phases.	Affecting the endometrial microenvironment, ovulation and pregnancy, getting involved in the pathophysiology of menorrhagia.	(42, 65–68)

### 4.1 TGF-β expression in the endometrial tissue

Exfoliated endometrial tissue contains all three isoforms of TGF-β, which are expressed in the human endometrium in a stage-specific and cyclically regulated manner. TGF-β1 levels were significantly increased in serum, peritoneal fluid, peritoneal tissue, and ectopic endometrial tissue in patients with endometriosis compared with normal women (44). The peritoneum, particularly the peritoneal mesothelium, is a source of TGF-β1. Studies have observed that women with endometriosis had higher levels of TGF-β1 mRNA in the peritoneum at sites adjacent to endometriosis lesions compared to distant sites (45, 46). Interestingly, one study found lower levels of TGF-β1 expression in ectopic endometrium, which may be due to post-transcriptional regulation of TGF-β1 influenced by the different microenvironments of the endometrium (47). Furthermore, TGF-β1 is found in the glandular cells, macrophages, and stromal cells of endometrial tissue (48, 49). While both endometrial and endometriotic cells secrete TGF-β1, stromal cells secrete higher levels of TGF-β1 than epithelial cells. On the other hand, compared to normal endometrial cells, endometriotic stromal and epithelial cells secrete higher levels of TGF-β2 (50). Moreover, a clinical study reported that patients with endometriosis had higher levels of TGF-β2 in their peritoneal fluid compared to patients without the condition (51). Researchers examined the three isoforms of TGF-β in the serum and peritoneal fluid of endometriosis patients and found overall high levels of TGF-β. The level of TGF-β1 in peritoneal fluid is higher than that in serum. In both serum and peritoneal fluid, the level of TGF-β3 was the lowest compared with the other two subtypes, while the level of TGF-β2 was comparable to that of TGF-β1 (52). During the menstrual cycle, three distinct expression profiles were observed. TGF-β1 shows a slight increase during the menstrual phase and remains relatively stable thereafter. It maintains a baseline level of regulation throughout the cycle, which ensures essential cellular functions such as proliferation and apoptosis. TGF-β2 exhibits a sharp five-fold increase from the early secretory phase to the secretory and menstrual phases, followed by a rapid five-fold decrease from the menstrual to the proliferative phase. This indicates a tightly regulated temporal expression of TGF-β2, which is crucial for the cyclical changes the endometrium undergoes. TGF-β3 mRNA levels increase threefold from the secretory phase to the menstrual phase and remain elevated throughout the proliferative phase. It likely contributes to the regulated proliferation and migration of endometrial cells, ensuring the proper reconstruction of the endometrial lining after menstruation (53). The various isoforms of TGF-β throughout the menstrual cycle underscore their specialized and potentially complementary roles in regulating endometrial physiology. Understanding these patterns can provide deeper insights into the complex regulatory mechanisms governing endometrial function, potentially opening new avenues for therapeutic interventions in conditions such as EMS and infertility.

### 4.2 Activin and inhibin expression in endometrium

An earlier study showed that the expression levels of the hormones inhibin A, inhibin B, and activin A were elevated in the peritoneal fluid of endometriosis patients throughout the menstrual cycle (54). Subsequent studies have shown that the concentrations of inhibin A and activin A in the cystic fluid of ovarian endometriosis patients were significantly higher than in peripheral blood, and slightly higher than those in the peritoneal fluid. This indicates that inhibin A and activin A are produced locally in ovarian endometriosis (55). Activin βA and follicular inhibitory mRNA are also discovered to be locally expressed in the human endometrial epithelium. Follicular inhibin does not significantly increase during the secretory phase, while activin A is expressed by endometrial epithelial and stromal cells, and increases during this phase (56). Other studies showed that women with EMS exhibited higher levels of follicular inhibin mRNA expression during the secretory phase compared to the proliferative phase. Thus, malfunction of the activin pathway in EMS may contribute to the aberrant expression of follicular inhibin (57). However, serum levels of follicular inhibin are significantly elevated in women affected by endometriosis, distinguishing it from other benign ovarian cysts (58). Due to its sensitivity and specificity, follicular inhibin is expected to serve as a valuable clinical marker for ovarian endometriosis.

On the other side, it was demonstrated that there were no significant changes in serum activin A and follicular inhibin in SPE and DIE, suggesting low diagnostic accuracy for OE (59). In a recent study, ectopic ESCs and peritoneal fluid from EMS patients were found to have a markedly higher expression of follicle suppressor-like I (FSTL1) compared to normal controls. This increase offered a fresh viewpoint on the genesis of EMS and could be the result of increased angiogenesis and proinflammatory factor secretion (60). The increased expression of follicostatins suggested that it could be a novel target for future treatments and a potential diagnostic indicator for EMS. Later on, it was demonstrated that through experimental methods the activin signaling system was present in both ovarian endometriosis and normal endometrium, and that both conditions produced activin A rather than inhibin (61). Activin βA mRNA is predominantly expressed in stromal cells, exhibits lower expression in epithelial cells, and demonstrates increased expression during the secretory phase of the menstrual cycle. Conversely, both stromal and epithelial cells exhibited negligible levels of inhibin α mRNA (62). In the same investigation, metaphase stromal cells exhibited low levels of inhibin α expression and high levels of activin βA expression. Activin promotes the differentiation of extravillous trophoblasts (EVTs) and the secretion of MMP-2 during trophoblast invasion. MMP-2 production in the immobilized EVT population decreases concomitantly with an increase in inhibin in the intravascular trophoblasts, indicating strong immunoreactivity between activin and inhibin. Accordingly, maternal tissue transformation is the primary source of activin, which is involved in both trophoblast invasion and tissue remodeling. In contrast, it is possible that inhibin may reduce trophoblast invasion (62). The study demonstrated for the first time that the human endometrium expresses Nodal and Cripto, in addition to activin A and its receptor. This discovery expands the list of genes associated with activin that are known to be locally expressed in the endometrium and raises the possibility that endometrial abnormalities in women affected by endometriosis are influenced by the activin system (63). In endometriosis women, ectopic endometrium during the secretory phase may exhibit decreased inhibin α and increased follicular inhibitor mRNA expression, alongside impaired activin A expression. This impaired ecdysis could potentially affect embryo implantation (55). At every stage of the menstrual cycle, whether it is the proliferative or secretory phase, the healthy endometrium expresses mRNA for the muscle growth inhibitor and the inhibitor of inhibition (29). Some studies have found that the glandular epithelium and stroma of the endometrium contained inhibin α and its co-receptor β-glycan. Women affected by endometriosis exhibited aberrantly elevated expression of β-glycan and inhibin α mRNA during the secretory phase. However, it remained unclear whether endometriosis itself was the cause of these abnormal expressions (64).

### 4.3 BMP expression in endometrium

The stroma and epithelium of the ectopic endometrium both exhibit high levels of expression of bone morphogenetic protein 6 (BMP6). Also BMP6 expression contributes to the formation of an estrogen-rich microenvironment and is strongly correlated with the high expression of estrogen receptor alpha (65). Granulosa cells (cumulus cells) affected by endometriosis exhibit elevated follicle-stimulating hormone levels during the proliferative phase, while BMP15 expression is inhibited (66). And BMP6 expression is downregulated in the cumulus cells of endometriosis women (42). The concentration of BMP2 in the peritoneal fluid of endometriosis women is lower than that in healthy women (67). This finding may negatively impact the process of ecdysis in these women, potentially causing issues with conception and pregnancy. Moreover, endometriosis patients show a higher concentration of BMP7 in the peritoneal fluid. This may be due to heavy menstrual bleeding associated with increased expression of genes encoding BMP7 molecules (67). Other studies have shown that the expression of BMP7 in the endometrium of endometriosis patients is significantly increased, and the expression reaches its peak in both the proliferative and secretory phases (68).

## 5 Effects of TGF-β on endometriosis

### 5.1 Promotion of TGF-β on adhesion, invasion and proliferation of ESCs

**Figure 2:**
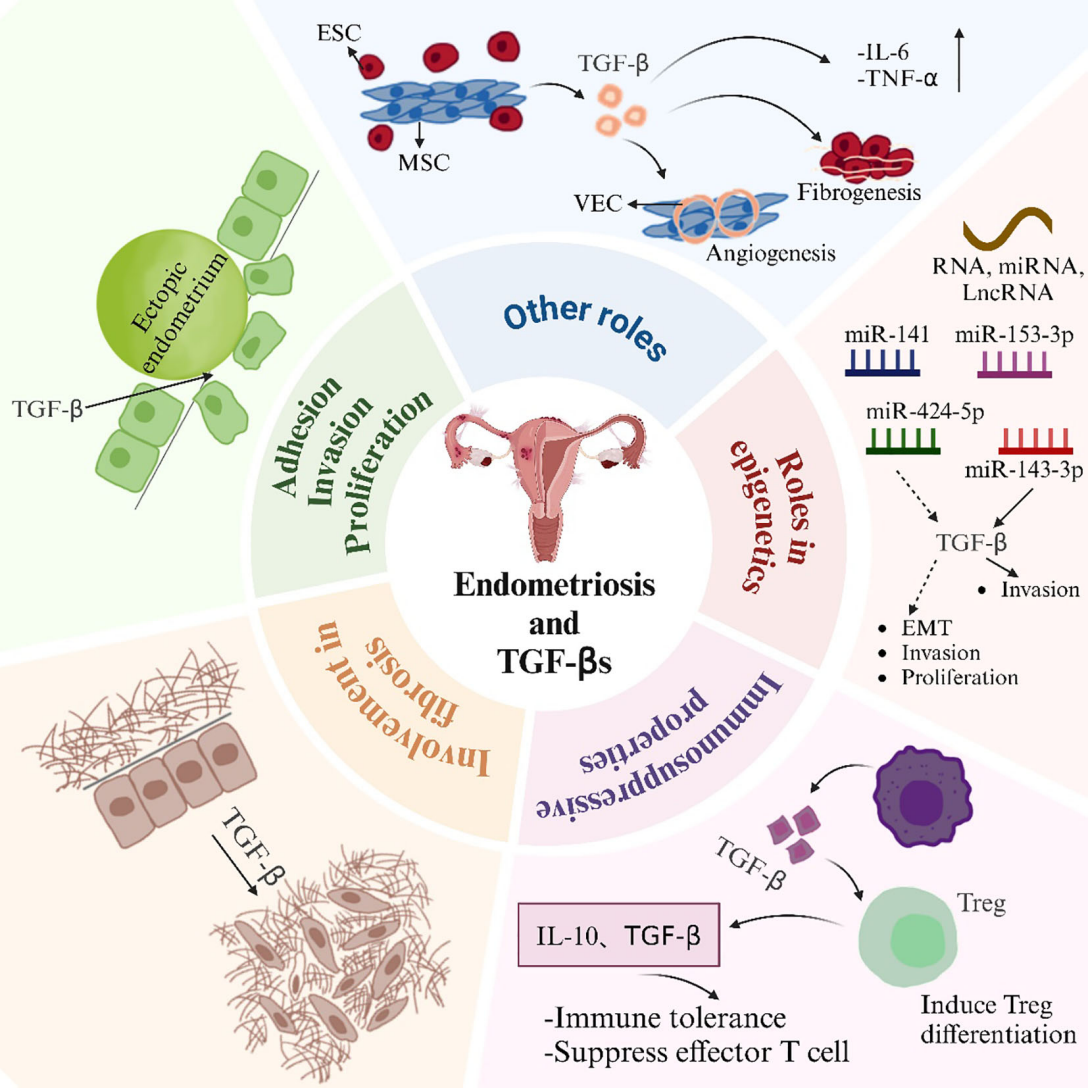
Effects of TGF-βs in EMS. ESC, endometrial stromal cell; MSC, mesenchymal stem cell; VEC, vascular endothelial cell.

An early hallmark of endometriosis involves the adhesion of endometrial tissue fragments to the pelvic mesothelium. This process is mediated by adhesion molecules and TGF-β1 (**Figure 2**). Research has revealed that endometriotic membrane epithelial cells express higher levels of TGF-β1 compared to normal endometrial cells. This higher expression facilitates more effective adhesion to mesothelial cells than that by normal epithelial cells. Moreover, TGF-β1 regulates αV, α6, β1, and β4 integrins, which directly promotes the adhesion of endometrial cells to mesothelial cells. The promotion occurs through the activation of the TGF-β1/TGF-βRI/Smad2 signaling pathway (69). Plasminogen activator inhibitor-1 (PAI-1) primarily inhibits fibrinolytic activity in the circulation. It is also involved in adhesion, migration, signaling, and the prevention of apoptosis (50). Researchers found that TGF-β1 or TGF-β2 stimulation of the endometrium and endometriotic cells boosted the cells’ Smad-dependent secretion of PAI-1. Meanwhile, they found that endometriotic cells secreted more PAI-1 compared to normal endometrial cells. This increased secretion enhanced cell adhesion and promoted the development of endometriosis (50). It has also revealed that a hypoxic microenvironment induces ESCs to generate excess TGF-β. This condition triggers the TGF-β1/Smad signaling pathway. As a result, integrin expression and adhesion of ESCs are improved (70, 71). Additionally, TGF-β1 promotes α2–6 sialylation. This modification strengthens the adherence of endometrial cells to the mesothelium. It has been shown that injecting NeuAcα2-6Galβ1-4GlcNAc reduces the formation of TGF-β1-induced endometriotic lesions. This injection also inhibits endogenous sialic acid binding (72). It has been demonstrated that TGF-β1 causes the acidification of endometrial cells. This acidification promotes the attachment of endometrial cells to the peritoneum. Thus, this process aids in the spread of endometriosis (72). TGF-β1 enhances the ability of human endometriotic cells to migrate, invade, and colonize. Studies have shown that TGF-β1 stimulates the migration of endometriotic cells by amplifying the integrin and FAK signaling axis. TGF-β1 enhances the adhesion of ectopic endometrial cells in the peritoneal region through the RHOGTPase signaling cascades and calcineurin-mediated migration (73).

TGF-β enhances the migration and invasion of ESCs. A theoretical foundation for the creation of novel therapeutic strategies aimed at blocking the TGF-β-ERK/MAPK signaling pathway in the prevention of endometriosis was established. This foundation was based on the demonstration that TGF-β overexpression boosted the migration and invasion of ectopic endometrial cells via this pathway (39). Ectopic endometrial tissue exhibits high levels of neurofibrillary protein 2 (NRP2) expression. It was discovered that the depletion of NRP2 restricted the migration, invasion, and epithelial-mesenchymal transition (EMT) of ectopic endometrial mesenchymal cells. TGF-β signaling activated SMAD2, which led to the transcriptional upregulation of NRP2 expression in these cells. This upregulation facilitated their migration and invasiveness (74). Endometriosis patients exhibit aberrant expression of vascular cell adhesion molecule 1 (VCAM-1). It was discovered that the knockdown of VCAM-1 could prevent the TGF-β-induced proliferation, migration, and invasion of endometriotic cyst stromal cells (75). It has been revealed that endometriotic cysts and the invasiveness of endometrial cells are regulated by the proteoglycan co-receptors SDC1 and SDC4 (76). SDC1, through TGF-β signaling, has been shown to control the invasive potential of endometriotic cells. This highlights the specific role of SDC1 in regulating cell invasion (77). Protein phosphatase Mg2+/Mn2+-dependent 1A (PPM1A) undergoes increased ubiquitination when tripartite motif (TRIM) 59 is overexpressed in endometriosis. TRIM59 facilitates the invasion of ectopic ESCs in endometriosis. This occurs through the inhibition of PPM1A via ubiquitination. And the activation of the TGF-β/Smad pathway is involved in this process (78). It is especially noteworthy that di(2-ethylhexyl) phthalate (DEHP) stimulates human endometrial and endometriotic cell proliferation, migration, and inflammatory responses by activating the TGF-β/Smad signaling pathway. DEHP induces EMT and stemness (79). Targeted inhibition of TAK1 was found to abolish the non-classical TGF-β1 signaling axis NFκB/Smad7. This inhibition resulted in the suppression of human endometriotic cell proliferation. Furthermore, it induced cell death involving autophagy (41). Studies have shown that beta-sitosterol can inhibit TGF-β-induced phosphorylation of Smads by regulating Smad7. This inhibition leads to a reduction in endometrial cell proliferation. Consequently, beta-sitosterol alleviates endometriosis (80).

### 5.2 Involvement in fibrosis

Fibrotic tissue is prevalent in ectopic endometriosis lesions. Smooth muscle metaplasia (SMM) is also commonly found in these lesions. TGF-β1 is a key mediator of fibrosis, which plays a crucial role in promoting fibrotic processes (**Figure 2**). TGF-β1 is produced by endometriotic ectopic cells in endometriosis. This protein causes fibrosis and ovarian tissue adhesion. It mediates these effects via the Smad2/3 signaling pathway (81). Researchers found that ovarian endometriotic tissues had higher levels of phosphorylated NR4A1 than normal endometrium. This observation was made when NR4A1 protein levels were measured in human endometrium and endometriotic tissues. TGF-β is the most potent promoter of fibrosis in endometriosis. This has been demonstrated by the phosphorylation of NR4A1. The phosphorylation occurs through prolonged stimulation in an AKT-dependent manner (82). This enhancement subsequently increased the expression of fibrotic markers. In endometriosis, activated platelets stimulate the TGF-β/Smad signaling pathway and release TGF-β1. This process promotes the EMT. It induces fibroblast-to-myofibroblast transdifferentiation (FMT). Fibrosis is the end result of this process. This process also increases cellular contractility and collagen synthesis (83). Wounds that are repeatedly damaged and healed are known as endometriotic lesions. Studies showed that TGF-β promoted wound healing in human endometriotic epithelial cells and endometriosis. It did this by controlling the shedding of beta glycans from these cells (84). Some studies suggested that Clostridium difficile might play a role in the development of EMS in the ovary. Transglutaminase (TAGLN)-positive myofibroblasts, which have the capacity to proliferate, adhere, and migrate *in vitro*, are created when quiescent fibroblasts are activated. This activation occurs through TGF-β signaling. The signaling is a result of Clostridium difficile infection of endometrial cells. It facilitates the onset of endometriosis (85).

### 5.3 Immunosuppressive properties

The pathogenesis of EMS is closely associated with an imbalance in immune regulation, which is characterized by both systemic and localized immune dysfunction (86). TGF-β is a pivotal immunomodulatory factor that maintains immune tolerance and contributes to inflammatory responses, with its effects determined by the cellular and cytokine interactions within the local microenvironment (87). In the peritoneal microenvironment of EMS, regulatory T cells (Tregs) and M2-polarized macrophages are increased and serve as major sources of TGF-β, thereby establishing an immunosuppressive environment that facilitates immune evasion and supports the survival of ectopic endometrial cells (88, 89). TGF-β induces the differentiation and expansion of CD4^+^CD25^+^FoxP3^+^Treg cells, thereby suppressing effector immune responses and promoting immune tolerance (90, 91). Elevated levels of TGF-β, interleukin-10 (IL-10), and cysteine-cysteine motif chemokine ligand 20 (CCL20) in peritoneal fluid act synergistically to activate regulatory Tregs, thereby suppressing effector T cell function and weakening immune surveillance (92, 93). In addition, macrophages and regulatory Tregs establish a bidirectional regulatory circuit, in which macrophage-derived CCL17 and CCL22 recruit Tregs, while Treg-secreted TGF-β cooperates with pro-inflammatory mediators to activate signaling pathways that promote angiogenesis and lesion progression (94). Macrophages can independently induce angiogenesis via direct secretion of TGF-β (95). M2-type macrophages and platelets also contribute to fibrosis by secreting TGF-β, a key profibrotic cytokine (96). TGF-β indirectly regulates Tregs differentiation by activating macrophages, which subsequently secrete IL-2, collectively contributing to the maintenance of immune tolerance (97). These findings suggest that targeting the TGF-β signaling pathway may restore macrophage function and offer a promising therapeutic avenue for endometriosis.

TGF-β functions as a major negative regulator of NK cell activity. In endometriosis, it facilitates immune escape and lesion progression by inducing high IDO expression in NK cells, suppressing cytotoxic receptor expression, and enhancing IL-10 secretion, thereby compromising NK cell-mediated cytotoxicity against ectopic endometrial cells (98). In the peritoneal cavity of patients with endometriosis, platelet activation leads to the release of TGF-β1, which suppresses NK cell cytotoxicity and facilitates local immune evasion by downregulating the NKG2D receptor. Notably, blockade of TGF-β1 reverses these immunosuppressive effects (99, 100).

Beyond Treg cells, macrophages, and NK cells, additional immune cell populations such as mast cells, neutrophils, monocytes, and B cells have also been implicated in TGF-β-driven immunopathological mechanisms. In endometriosis, TGF-β promotes fibrosis, mast cells activate the NLRP3 inflammasome pathway, and neutrophil infiltration aggravates oxidative stress within ectopic lesions. Fexofenone has been shown to attenuate disease progression by concurrently suppressing TGF-β expression, mast cell activation, and neutrophil recruitment (9, 101). E2 promotes the recruitment of mast cells to endometriotic lesions by upregulating TGF-β expression in ectopic endometrial cells. Moreover, E2 directly induces mast cell degranulation, leading to the release of mediators such as nerve growth factor (NGF), which contributes to neuronal sensitization and endometriosis-associated pain. These findings highlight TGF-β as a critical mediator linking estrogen signaling to mast cell activation (102). Moreover, monocytes regulate their own proliferation, aggregation behavior, and adhesion molecule expression via autocrine TGF-β1 (103). Abnormalities in the TGF-β signaling pathway and B cell-mediated autoimmune responses have been observed in endometriosis. Peritoneal fluid shows significant enrichment of auto antibodies targeting TGF-β pathway components and B cell-associated proteins, providing new perspectives for disease classification and immune-targeted therapies (104).

### 5.4 Roles in epigenetics

The cellular process known as the EMT occurs when polarized, immobile epithelial cells interact with membrane structures, such as adhesions and gap junctions. This interaction causes them to transform into invasive and migrating mesenchymal cells (105). Several signals, including TGF-β signaling and estrogen stimulation, can induce EMT in endometriosis (**Figure 2**) (106). It has been shown that the aberrant expression of microRNA (miRNA) may be linked to the onset of endometriosis (107). And by inhibiting the TGF-β1/SMAD2 signaling pathway in endometriosis, microRNA-141 suppressed the proliferative and invasive capacities, as well as the epithelial-to-mesenchymal transition induced by TGF-β1 (108). Furthermore, LncRNA AFAP1-AS1 regulated the growth and apoptosis of endometriotic cells by activating the STAT3/TGF-β/Smad signaling pathway through miR-424-5p (109). By interacting with the miR-153-3p/TMSB4X axis, CircPIP5K1A activated the TGF-β signaling pathway, thereby accelerating the development of endometriosis (110). Also, miR-143-3p targeted VASH1 to activate TGF-β signaling, thereby promoting the invasion and migration of endometriotic stromal cells (111).

### 5.5 Other roles

TGF-β can encourage the development of new blood vessels by controlling the expression of angiogenic factors. This regulation is crucial for angiogenesis. TGF-β has been shown to upregulate vascular endothelial growth factor (VEGF) expression in endometriosis patients. This upregulation encourages the development of aberrant blood vessels. In diseased tissues, these aberrant blood vessels create a dense vascular network, which exacerbates the ischemic and hypoxic conditions of the tissues (112). TGF-β can stimulate inflammatory reactions by controlling the expression of genes linked to inflammation. This regulation plays a crucial role in the inflammatory process. The increased expression of TGF-β in endometriosis tissues is associated with the infiltration of inflammatory cells. It leads to the upregulation of pro-inflammatory factors like interleukin-6 (IL-6) and tumor necrosis factor-alpha (TNF-α). These inflammatory reactions further lead to the development of tissue fibrosis and pain (7).

The pathophysiology of endometriosis also involves endometrial mesenchymal stem cells (MSCs). It has been found that endometrial mesenchymal stem cells activate the Wnt/β-catenin pathway through paracrine TGF-β1 and Wnt1, enhancing interstitial cell fibrosis in ovarian endometriosis (113). Fibrosis in endometriosis is promoted by TGF-β1 secreted by endometrial MSCs. This process occurs via SMAD3/DNMT3A-mediated RASAL1 inhibition (**Figure 2**) (114).

## 6 Effects of activin and inhibin on endometriosis

**Figure 3:**
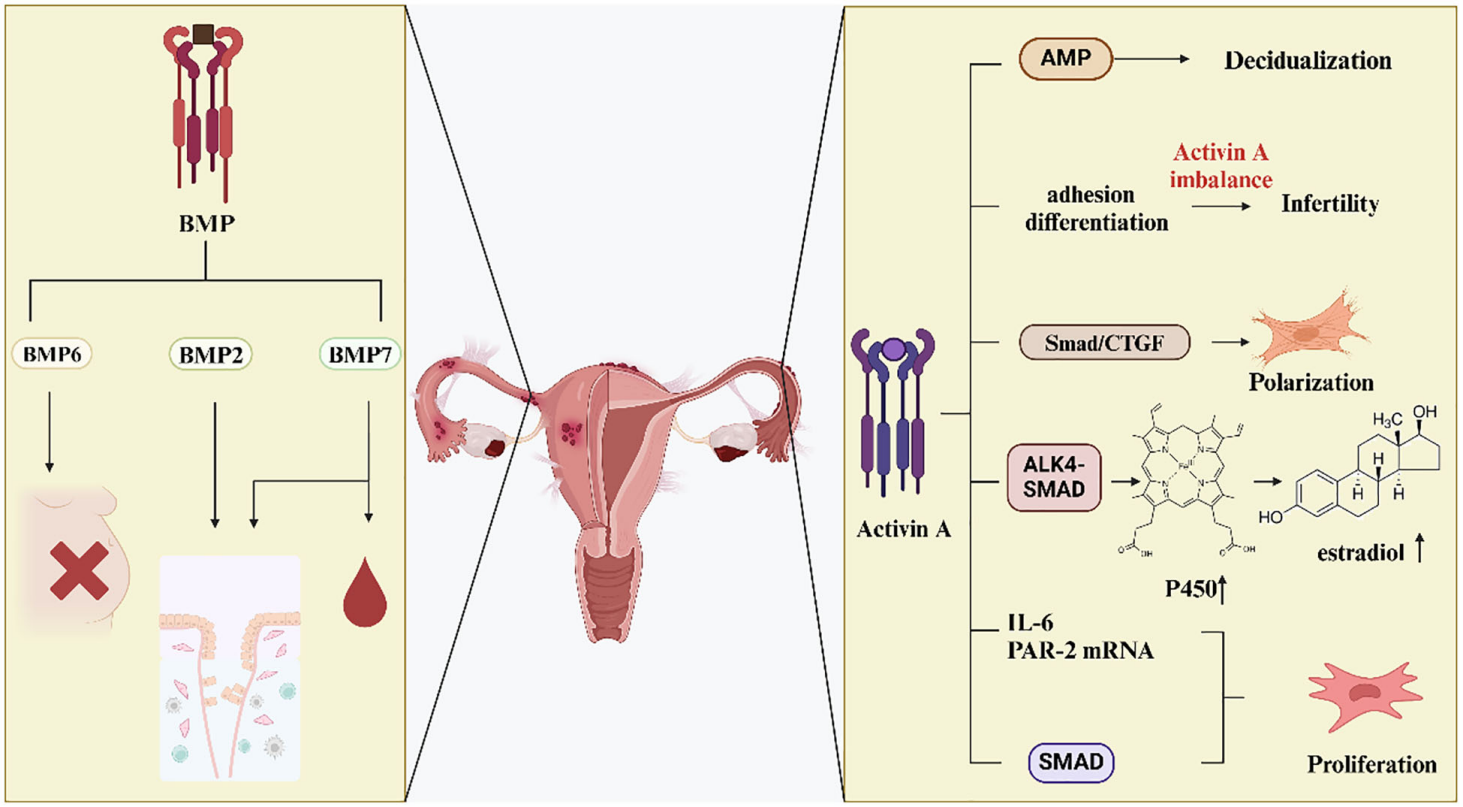
Effects of activin and BMP in EMS. Decreased BMP6 expression may lead to infertility. Altered expression of BMP2 and BMP7 affects endometrial metamorphosis. In addition, alterations in BMP7 regulate menstrual bleeding.Activin A affects the adhesion, differentiation and proliferation of ESCs through various signaling pathways, which in turn affects their fibrosis and the development of endometriosis.

Inhibin and activin both impact the growth of endometriosis lesions (**Figure 3**). Studies have shown that activin A uses the cyclic AMP signaling pathway, which causes human ESCs to undergo metamorphosis *in vitro*. Activin A has the potential to function as a local growth factor. It regulates endometriotic cells through autocrine-paracrine mechanisms. These mechanisms include the regulation of proliferation, differentiation, and apoptosis (63). Reports of activin receptor expression on the cell surface and in the cytoplasm supported this finding. Smad2, Smad3, and Smad4 proteins were expressed in the cytoplasm and nucleus of normal endometrium and ovarian endometriosis tissues. This expression indicates that these tissues are targets of activin A’s autocrine-paracrine action. And both ovarian endometriosis tissues and normal endometrium have an active activin signaling system (61).

In addition, activin A was found to affect SMAD7, resulting in up-regulation of its expression. It reveals that activin A stimulates the SMAD signaling pathway and encourages the formation of endometriotic lesions (115). Activin A controls embryonic trophectoderm adhesion and differentiation and also causes ecdysis in cultured ESCs (54). Afterwards, the study examined the hypothesis that dysregulation of local activin A levels causes reduced trophoblast cell adhesion and fewer adhesion molecules to be produced. This dysregulation ultimately leads to the failure of ectopic implantation. For women with endometriosis, this could be a potential cause of infertility (116). Another study found that during the proliferative and secretory phases of endometriosis patients’ endometrium, there was a decrease in the expression of activin A, Cripto, inhibin α, and follicular repressor mRNA. This decrease may cause infertility in women affected by endometriosis (55). Researchers investigated the control and role of activin A in endometriotic stromal cells. They conducted *in vitro* experiments and discovered that activin A, when induced by TNF-α or IL-1β, may increase the proliferation of endometriotic stromal cells. Additionally, it stimulated the expression of IL-6 and PAR-2 mRNA, thereby potentially promoting endometriosis (117).

Activin A is an endometrial secretory product implicated in angiogenesis and inflammation. The inflammatory response and aberrant tissue remodeling linked to endometriosis may be caused by activin (118). It suggests that elevated levels of activin can contribute to the chronic inflammation and abnormal tissue growth observed in endometriosis patients, highlighting its potential as a therapeutic target for managing and treating this condition. Follicular inhibin is a binding protein for activin A. Previous research revealed that follicular inhibin expression was abnormal in ovarian endometriosis lesions. This abnormal expression pattern suggests a potential disruption in the regulatory mechanisms governing follicular inhibin in these lesions. Further studies needed to understand the implications of this dysregulation on the pathophysiology of ovarian endometriosis (60). This may contribute to altered effects of activin A on angiogenesis, and impact endometrial differentiation. Understanding these changes is crucial for elucidating the role of activin A in these processes. In endometriosis, activin A inhibits the release of IL-8 and vascular endothelial growth factor (VEGF) in ESCs. This inhibitory effect is mitigated by follicular inhibin (119). IL-1β and TNF-α in endometriotic stromal cells also induce the expression of follicular inhibin. Although follicular inhibin is present in endometriosis cystic fluid, activin activity remains predominant *in vivo*. This observation suggests that the regulatory influence of activin surpasses that of follicular inhibin in the pathological environment of endometriosis. Further research is required to understand the mechanisms behind this predominance and its implications for endometriosis progression and treatment (120).

Estrone is synthesized from circulating androstenedione through the catalytic action of the enzyme aromatase P450. This enzyme is predominantly located in skin and adipose tissue. The conversion process highlights the role of peripheral tissues in steroidogenesis, particularly in the production of estrogenic compounds such as estrone. The positive feedback loop in steroidogenesis leads to the overexpression of key steroidogenic genes. As a result, androstenedione in circulation disseminates to the endometrial tissue, where it undergoes conversion into estradiol. This process underscores the intricate regulatory mechanisms involved in endometrial steroidogenesis. The positive feedback loop enhances the secretion of TGF-β in endometrial tissue, and increases local estradiol production. These effects highlight the interconnected regulatory mechanisms that drive endometrial function and pathology (121).

Extensive research has demonstrated that activin A stimulates the expression of aromatase P450 via the ALK4-Smad pathway. This stimulation, in turn, promotes the development and survival of endometriosis focal tissue. And it enhances the secretion of estradiol in ESCs. These findings underscore the critical role of activin A in the pathophysiology of endometriosis (122). Apart from the ALK4-Smad route, the activin A aromatase P450 promoter II is bound by activated Smad3 proteinin ESCs. This binding increases the transcription of aromatase P450 in endometriosis. These suggest that an alternative regulatory pathway by which activin A contributes to the pathophysiology of endometriosis (123).

Activin A stimulates the differentiation of endometrial MSCs into myofibroblasts via the Smad/CTGF pathway, which is dependent on STAT3. These findings highlight a novel mechanism through which activin A influences endometrial fibrosis and the progression of endometriosis (124). It indicates that inhibiting the activin A pathway hampers the differentiation of endometrial MSCs into myofibroblasts. This inhibition subsequently reduces endometriosis-related fibrosis. Under hypoxic conditions, endometrial cells can undergo EMT induced by prostaglandin E2 (PGE2) and thrombin. Experimental evidence has shown that PGE2 and thrombin induce myofibroblast differentiation in ESCs via activin A and connective tissue growth factor (CTGF). It promotes EMT and significantly influences fibrosis in endometriosis. These results underscore the critical role of cellular plasticity and signaling pathways in the pathophysiology of endometriosis (125). The expression of TGF-β type III receptor is observed in endometrial glandular cells and endothelial cells. This receptor is also known as β glycan (BG). Under normal conditions, these cells shed their extracellular structural domains, releasing soluble BG. This soluble form inhibits the transduction of TGF-β signals. In the present study, activin A inhibited the shedding of BG while simultaneously increasing BG mRNA expression in endometriotic cells. This regulation occurred through the ALK4-SMAD3-dependent pathway. It suggests that activin A plays a crucial role in modulating β glycan dynamics and TGF-β signaling in endometriotic cells (25).

## 7 Effects of BMP on endometriosis

BMP is a multifunctional extracellular growth factor involved in various cellular processes (**Figure 3**). Both the endometrial and maternal-fetal interfaces express BMP ligands, receptors, and associated transduction molecules. BMP signaling plays a crucial role in modulating the interactions between endometrial and maternal-fetal tissues. These interactions are essential for successful implantation and the maintenance of pregnancy. Dysregulation of BMP ligands, their receptors, or associated signaling pathways can lead to altered endometrial remodeling, potentially resulting in obstetrical complications or infertility. In other words, the process of metamorphosis is influenced by BMPs. Among these, BMP2 is a growth factor that plays a critical role in this process (126). In individuals with endometriosis, decreased BMP signaling was found to hinder endometrial metamorphosis. This impairment in signaling disrupts the normal cellular and tissue transformations necessary for a healthy endometrial environment. BMP2 supplementation increases the capacity for metamorphosis in stromal cells and endometrial assemblies of these patients. It shows that BMP2 plays a crucial role in enhancing cellular and tissue transformations (127). A variety of cell types, including pericytes that support the neovascular basement membrane structure, are regulated in terms of proliferation and differentiation by the pleiotropic BMP2 signaling molecule (67). Women affected by endometriosis had lower concentrations of BMP-2 in their peritoneal fluid. This reduction disrupted the normal structure of blood vessels and encouraged the development of endometrial fibrosis and adhesions. In the same study, increased concentrations of BMP-7 were found to control menstrual bleeding. This regulation, in turn, encouraged the development of endometrial implants (67). Overall, the formation of the endometrium appears to be influenced by both BMP-2 and BMP-7. Previous studies showed that BMP-6 was highly expressed in endometriosis. This elevated expression suggests a potential role for BMP-6 in the pathophysiology of endometrial disorders (65). Subsequent research found that the expression of BMP-6 and SMAD4 was reduced in women with peritoneal endometriosis. Additionally, it was found that granulosa cell function may be altered in women with endometriosis, potentially affecting their fertility (42). The BMP7-SMAD4-CDH1 signaling pathway may be negatively regulated by miR-542-3p. CDH1 mRNA transcripts, as well as SMAD4 and BMP7 mRNA transcripts, were down-regulated. Conversely, the expression of miR-542-3p was highly elevated. According to these results, women with endometriosis do not exhibit the typical endometrial epithelial phenotype. This finding supports the hypothesis that alterations in epithelial characteristics play a significant role in the etiology of endometriosis (68).

## 8 Emerging TGF-β-targeting therapeutic strategies

The possible involvement of endometrial MSCs in endometriosis has also been a significant area of recent study. Stem cell therapy is considered a cutting-edge therapeutic approach for treating fibrosis and uterine adhesions. This innovative treatment has shown potential in regenerating damaged tissues and improving uterine function. Studies have shown that TGF-β1 is secreted by endometrial MSCs which are a pro-fibrotic factor that stimulates the fibrotic process in endometriosis. This stimulation occurs through pathways such as Wnt/β-catenin or SMAD3/DNMT3A (7, 114). But the correlation between the two remains unclear. Furthermore, miRNAs impact the EMT and the TGF-β signaling pathway. These interactions in turn influence the development of endometriosis. miRNAs also impact the proliferation, apoptosis, migration, and invasion of endometriotic stromal cells. Therefore, a thorough understanding of TGF-β superfamily members and their interactions with different pathways is necessary to fully realize their therapeutic potential. In the future, these targets might become viable therapeutic objectives for treating endometriosis.

So far, several medications have been discovered to influence TGF-β expression and reduce the size of endometriotic lesions in endometriosis (**Table 3**). For instance, kiwi root extract regulated TGF-β expression in endometriotic lesions by downregulating VEGF-A via TGF-β1, and prevented neovascularization in endometriosis (140). Resveratrol exhibited antioxidant, anti-inflammatory, and anti-angiogenic properties. It could enhance the advancement of endometriosis by reducing TGF-β expression in ESCs. However, further investigation was required to determine the precise mechanism of action (128). Cannabidiol impacted endometriosis by inhibiting fibrosis and downregulating TGF-β expression in rat endometriotic cells (129). Fisetin reduced fibrosis in endometriotic lesions and decreases TGF-β expression in endometriotic cells (101). Salbutamol could lower the expression of TGF-β in lesions and lessen the content of collagen fibers, showing an obvious inhibitory effect on fibrosis (141). These studies suggest that TGF-β-targeting medications may be used to treat endometriosis. Notably, our understanding of the precise mechanism of action of these agents is limited. Future research should focus on their relevant mechanisms to explore more clinical potentials.

**Table 3:** Therapeutic effects of drugs on EMS via TGF-β.

Medication	Experimental Models	Mechanism of Action	Role of TGF-β	Therapeutic Effect	Side Effects	Reference
Resveratrol	HESCs	The inhibition of VEGF, TGF-β, and MMP-9 expression reduces the generation of ROS and RNS by suppressing the NF-κB and MAPK pathways.	Blocking TGF-β transcription reduces TGF-β-induced fibrosis and cell proliferation.	Reducing VEGF, TGF-β, and MMP-9 expression, and inhibiting lesion progression and angiogenesis in patients with endometriosis.	Minor gastrointestinal discomfort	(128)
CBD	Rats	Inhibiting lipid peroxidation, reducing ROS generation, and restoring endogenous antioxidant defence mechanisms with antioxidant, antifibrotic, and anti-inflammatory properties.	Downregulation of TGF-β expression reduces fibrosis and the inflammatory response, while improving lesion morphology.	A significant reduction in lesion diameter, volume, area, pain, and inflammatory response in rats with endometriosis.	No significant side effects	(129)
Fisetin	Rats	Regulating the NLRP-3 inflammasome pathway and oxidative stress, reducing neutrophil infiltration, inhibiting the NF-κB pathway, increasing Bax and caspase-3 expression, decreasing Bcl-2 expression, and promoting apoptosis.	Reducing TGF-β expression, inhibiting fibrosis and lesion expansion, and attenuating endometriosis-induced inflammation and oxidative stress.	Reducing the volume and fibrosis of endometriosis lesions, decreases the levels of oxidative stress markers and inflammatory factors, and promoting the apoptosis of lesion cells.	No significant side effects	(101)
Neferine	Mice and 12Z cell	Reducing the expression of fibrosis-related proteins, such as α-SMA, Col-1, CTGF, and FN.	Inhibition of fibrosis by suppressing TGF-β/ERK signaling pathway.	Reducing fibrosis in endometriosis and inhibiting the proliferation, invasion, and migration of 12Z cells.	No significant side effects	(130)
Protopanaxadiol (PPD)	Mice and HESCs, U937 cells, NK cells	Reducing the inflammatory response, enhancing the expression of dysplasia-related genes, and promoting the proliferation and function of dysplastic NK cells through the up-regulation of endometrial tolerance-related genes.	Enhancing embryo implantation and pregnancy maintenance, reducing endometriosis-induced infertility and miscarriage, through the promotion of TGF-β expression.	Increasing the pregnancy rate and the number of implanted embryos, reducing the risk of miscarriag.	No significant risk of osteoporosis or other serious side effects compared to GnRHa.	(131)
HES5	Mice and HESCs	Inhibiting FBXW7 expression, reducing TGIF1 degradation, suppressing activation of the TGF-β signaling pathway, and promoting apoptosis through HES5 up-regulation.	Reducing Smad2 phosphorylation, and inhibiting the TGF-β signaling pathway, leading to the attenuation of endometriosis through the down-regulation of TGF-β1 and PAI-1 expression,.	Inhibiting lesion development and attenuating pathological changes.	No significant side effects	(132)
STAT3 inhibitor	Mice	Reducing IL-6-mediated signaling and the production of pro-inflammatory cytokines through inhibition of the JAK/STAT signaling pathway.	Reducing fibrosis and lesion expansion through the inhibition of TGF-β expression.	Reducing the size of endometriosis lesions, particularly after weeks 2 and 3.	No significant side effects	(133)
Resveratrol	HGCs	Inhibiting Bax and Caspase 9 expression, increasing expression of Bcl-2, attenuating apoptosis through the mitigation of oxidative stress.	Inhibiting apoptosis and protecting ovarian function by reducing TGF-β expression	Improving ovarian function and potentially enhances fertility through the reduction in the rate of apoptosis of ovarian granulosa cells.	No significant side effects, but inducing cellular oxidative stress by high doses.	(134)
Allium cepa	Rats	Reducing the expression of the proliferation marker Ki67, with anti-inflammatory, antioxidant, and antifibrotic properties.	No change of TGF-β1 and α-SMA levels, indicating a limited effect on fibrosis.	Reducing the proliferative potential of endometriotic lesions, but minimal effect on fibrosis.	No significant side effects	(135)
Rubus idaeus Polyphenols Extract	Rats	Inhibitiing MMP-2 and MMP-9 expression, with the anti-inflammatory and antioxidant properties.	No obvious affect on TGF-β1 expression, but inhibiting fibrosis and lesion extension by reducing MMP levels.	Reducing the diameter of endometriotic lesions and lowering the inflammation levels.	No significant side effects	(136)
HYSJ-EL	Rats	Inhibiting the inflammatory response, peripheral nerve sensitization, and pelvic adhesions, reducing pain and focal fibrosis by down-regulating the expression of PGE2, IL-6, TNF-α, and MIP-2.	Reducing TGF-β expression and inhibiting fibrosis and adhesion formation, resulting in pain relief.	Increasing the thermal pain threshold, reducing lesion volume and fibrotic area, and decreasing inflammatory factor levels.	No significant side effects	(137)
Baicalein	Mice and HESCs	Reducing the expression of MMP-2, MMP-9, and MT1-MMP through the inhibition of FURIN-MT1-MMP-mediated cell invasion, thereby inhibiting endometriosis through an anti-invasive mechanism.	Reducing TGFB1 secretion and inhibiting TGFB1-induced FURIN expression, thereby decreasing MT1-MMP activation and the cell invasion ability.	Reducing the weight and number of lesions and decreasing the expression of TGFB1, FURIN and MT1-MMP.	No significant side effects	(138)
Leflunomide	Rats	Inhibiting the progression of the G1/S phase of the cell cycle, interfering with pyrimidine biosynthesis, inhibiting protein tyrosine kinase activity, and reducing the production of autoantibodies and cytokines.	Reducing TGF-β1 expression, inhibiting fibrosis and lesion extension.	Reducing the volume of endometriosis lesions and TGF-β1 expression, demonstrating inhibition of lesion growth.	No significant side effects	(139)
Di-(2-ethylhexyl) phthalate(DEHP)	EEECs	Promoting cell proliferation, migration, stemness, and EMT, thereby increasing inflammatory and immune responses.	Activating the TGF-β/Smad signaling pathway, enhancing cell proliferation and migration.	Promoting the progression of endometriosis through enhanced proliferation and migration of EEECs.	Potential reproductive toxicity and other endocrine-disrupting effects.	(79)
Kiwi Root Extract	Mice	Inhibiting the expression of pro-inflammatory cytokines, including IL-6, IL-8, IL-1β, and TNF-α, reducing the expression of angiogenic factor VEGF-A, inhibiting COX-1 and COX-2 expression.	Reducing TGF-β1 expression, inhibiting TGF-β1-related signaling pathways, and decreasing fibrosis and lesion extension.	Reducing the volume of endometriosis lesions and TGF-β1 expression, demonstrating inhibition of lesion growth and a reduction in the inflammatory response.	No significant side effects	(140)
Salbutamol	Mice	Inhibiting angiogenesis and fibrosis as a β2-adrenergic receptor agonist, while increasing apoptosis in focal cells by reducing the expression of immune inflammatory cells and factors.	Reducing TGF-β expression and attenuating fibrosis and lesion extension.	Reducing the number, volume, and weight of endometriosis lesions, decreasing inflammation and fibrosis, promoting apoptosis of lesion cells, and lowering nerve growth factor expression.	No significant side effects	(141)
Cisplatin	Rats	Inhibiting cell proliferation and inducing apoptosis by binding to the DNA of target cells and promoting DNA cross-linking.	Reducing TGF-β expression and inhibiting fibrosis and lesion expansion.	Reducing the volume of endometriosis lesions and decreasing the expression of VEGF, P450arom, TGF-β, and MMP-2.	Potential nephrotoxicity and neurotoxicity	(142)
Letrozole	Rats	Inhibiting the growth of endometriosis lesions by suppressing aromatase activity and reducing estrogen production both systemically and locally as a third-generation aromatase inhibitor.	Adjusting TGF-β expression primarily by reducing estrogen.	Reducing the volume and histological scores of endometriosis lesions, and the expression of P450arom and VEGF.	Causing hyperandrogenemia, resulting in polycystic ovary syndrome-like symptoms and abnormal bone metabolism.	(143)

Additionally, the search for TGF-β-based treatment strategies for pathologies sharing key features with endometriosis may bring new insights. For instance, fibrosis is an important pathological feature of all types of endometriosis. Meanwhile, it is also a common pathological feature of pulmonary fibrosis, liver fibrosis, kidney fibrosis, systemic sclerosis and other fibrotic diseases, and is closely related to the occurrence of a variety of tumors (144). TGF-β stimulates the activation and proliferation of fibroblasts, leading to extracellular matrix deposition. Its increased expression can cause many fibrotic diseases, and its expression level is often related to the severity of the disease. Researchers have developed different strategies to regulate the activity of TGF-β based on its molecular mechanisms of signaling and activity, including TGF-β -targeted antibodies, small molecule receptor inhibitors, ligand traps, antisense oligonucleotides, etc (145). These results may be crucial for future development of targeted TGF-β therapy for endometriosis.

## 9 Conclusions and outlook

This study provided a comprehensive review of the role of TGF-β superfamily in endometriosis. The relationship between TGF-β, activin, inhibin and BMP and endometriosis was discussed, and the signal transduction and expression of these factors in endometrium were summarized. TGF-β can stimulate the adhesion, invasion and proliferation of ESCs, affecting the occurrence of endometriosis. It also plays a role in the development of fibrosis in the focal tissue of endometriosis, and suppresses the immune response. However, the precise function of TGF-β in controlling blood vessels still needs to be further clarified. Current knowledge about the involvement of TGF-β superfamily members in endometriosis underscores the great potential and complexity of this field. It underscores the necessity for comprehensive investigation into the involvement of TGF-β superfamily members in endometriosis. This includes their effects on endometrial metaplasia, focal tissue fibrosis, and cell migration and proliferation. To fully realize the potential of TGF-β superfamily members in clinical therapies, their complex roles in endometriosis should be continuously explored and elucidated.

## Data Availability

The original contributions presented in the study are included in the article/**Supplementary Material**. Further inquiries can be directed to the corresponding authors.
