# Supplementary material for: The role of TGF-β superfamily in endometriosis: a systematic review
Source: Front Immunol. 2025 Aug 12;16:1638604. doi: 10.3389/fimmu.2025.1638604 (PMC12378113; doi:10.3389/fimmu.2025.1638604)
Supplement: Supplementary file 1 [file DataSheet1.pdf]

# Supplementary materials

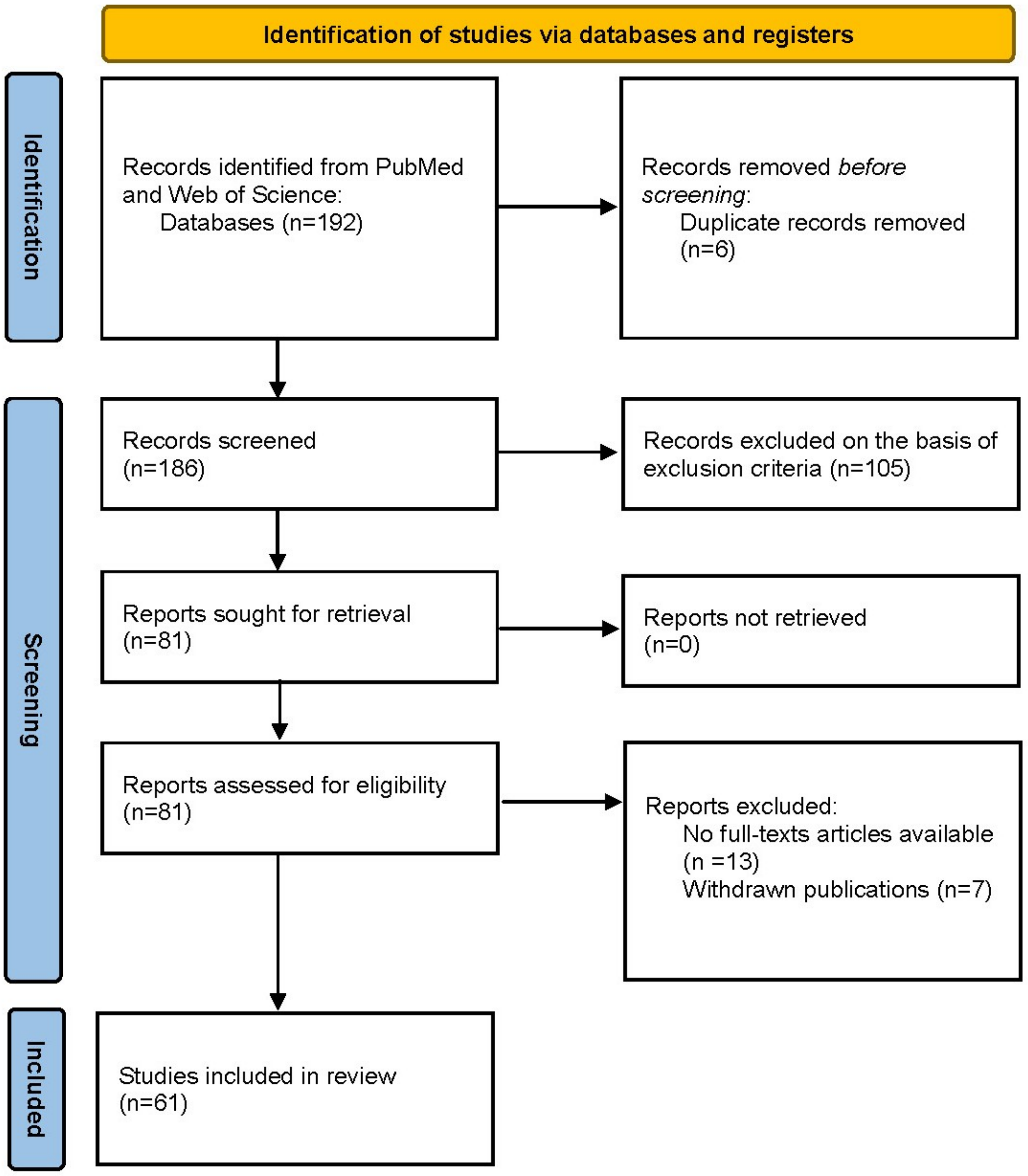


**Figure S1 PRISMA flow diagram for identifying the included studies.**

## Table S1 Summary and key findings of the included studies

| Author(s) | Journal | Key Findings |
|---|---|---|
| Lukanxuan Wu et al. | Reproductive Sciences | CD45+ cells are abnormally activated in the ectopic endometrium of mild/mild endometriosis.The expression of TGF-β1 in CD45+ cells is increased. TGF-β1 inhibits the expression and deindividualization of PR in ectopic ESCs. |
| Budi Santoso et al. | Journal of Reproductive Immunology | Serum GDF-15 might serve as a candidate biomarker for endometriosis severity. |
| Agnes N. Mwaura et al. | Biomolecules | Activin A suppresses the shedding of BG in human endometriotic cells via the ALK4-SMAD3 axis, whereas inhibin A also reduces BG shedding but independently of follistatin. |
| Cynthia Dela Cruz et al. | Reproductive Sciences | Proliferative endometrium of women with endometriosis shows a decrease in gene expression of Cripto and SMAD3 and no alterations in gene and protein analysis of Nodal, as well as in the immunostaining of pSMAD3 and SMAD4. |
| Patrizia Carrarelli et al. | Reproductive Sciences | The increased expression of myostatin in OMA, DIE, and endometrial cancer is corroborated by the increased expression of the type II receptors and at least 1 type I receptor, suggesting also an increased cellular response. |
| Zhihong Liu et al. | Experimental and Therapeutic Medicine | Overexpression of TGF-β enhances the migration and invasion of ectopic endometrial stromal cells (ESCs) via the ERK/MAPK signaling pathway. |
| Jyoti B Kaushal et al. | Cytokine | TAK1 is a key nodal player in TGFβ1–mediated aberrant activation of NFκB in endometriotic cells. TGFβ1-stimulated activation of NFκB-p65 transduced the expression of Cox2 and Smad7. |
| Emily De Conto et al. | Acta Obstetricia et Gynecologica Scandinavica | The expressions of BMP-6 and SMAD4 in women with peritoneal endometriosis are decreased compared with the control group. |
| Rinako Miura et al. | BMC Cancer | Nodal is a multifunctional cytokine involved that plays a key role in the biology of |

| | | |
|---|---|---|
| | | ovarian endometriosis-carcinoma lesions. |
| Vicky J Young et al. | PLoS One | The expression of several TGF-β signalling targets is altered within the peritoneum of women with endometriosis and these may play a role in lesion development. |
| Anna Zubrzycka et al. | International Journal of Molecular Sciences | The eutopic endometrium of women with endometriosis shows higher expression of the mRNA TGF-β1, SMAD3 and ILK, and the level of miR-21 does not change compared to the endometrium of healthy participants. In endometriosis, miR-21 may be an inhibitor of TGF-β1-SMAD3-ILK signaling. |
| M Cecilia Johnson et al. | Reproductive Biology and Endocrinology | An altered expression of c-myc, TGF-beta1 and bax is observed in eutopic endometrium from endometriosis, suggesting its participation in the regulation of cell survival in this disease. |
| Shin-ichi Komiyama, M.D. et al. | Journal of Reproductive Medicine | TGF-β1 activity is increased at sites of endometriosis due to enhanced production of both uPA and TGF-β1 by glandular epithelium and because plasmin activates TGF-β1 after being converted from plasminogen by uPA. |
| Cong Sui et al. | American Journal of Translational Research | Smad-dependent and TBR1-dependent ERK1/2 pathways are necessary for TGF-β-dependent high level secretion of PAI-1, which might increase cellular deadhesion. |
| Justyna Sikora et al. | Immunology Letters | In endometriosis, TGF-β could affect differentiation of T helper (Th) cells, hence produce more IL-17 and IL-10 to PF and might have an indirect influence on inflammation, which is associated with higher IL-1β and IL-6 levels. |
| Ana Luiza L Rocha et al. | Fertility and Sterility | The disturbed expression of endometrial activin A, cripto (activin receptor antagonist), and follistatin (activin-binding protein) suggests a dysfunction of the activin pathway in endometriosis. |
| Fernando M Reis, M.D. et al. | Fertility and Sterility | Inhibin A and activin A concentrations in the cystic fluid are slightly higher than in peritoneal fluid and significantly higher than in serum. Immunoreactive alpha and betaA subunits are strongly expressed both in the epithelial and stromal components of ovarian endometrioma. |

| | | |
|---|---|---|
| Sha-Ting Lei et al. | Society for Reproduction and Fertility | The elevated expression of FSTL1 may play a key role in accelerating the development of EMS via enhancing the secretion of proinflammatory factors and promoting angiogenesis. |
| P Florio et al. | Human Reproduction | Serum follistatin is increased in women with endometriosis and allows clear distinction between endometrioma and other benign ovarian cysts. Follistatin has the sensitivity and specificity to become a useful clinical marker of ovarian endometrioma. |
| Yasushi Mabuchi et al. | International Journal of Molecular Medicine | Activin A, but not inhibins, is produced by ovarian endometriosis and the normal endometrium, and that the activin signal transduction system exists in both ovarian endometriosis and the normal endometrium. |
| Paulo B Torres et al. | Reproductive Sciences | The altered endometrial expression of activin A and cripto during the menstrual cycle and the differences observed in the endometriotic tissue support the involvement of the activin system in endometrial changes of women with endometriosis. |
| Cynthia Dela Cruz et al. | Gynecologic and Obstetric Investigation | The expression of α-inhibin mRNA (not protein) and β-polysaccharide (mRNA and protein) is abnormally increased in the secretory endometrium of patients with endometriosis. |
| Joanna Janusz et al. | Biomedicines | BMP-2 and BMP-7 and their soluble receptors, ALK-1 and BMPR2, are involved in the formation of endometriosis. |
| Anna Zubrzycka et al. | International Journal of Molecular Sciences | The observed significantly high level of expression of miR-542-3p with simultaneous downregulation of BMP7, SMAD4, and CDH1 mRNA transcripts in the endometrial foci may be suggestive of the suppressive function of this miRNA in BMP7–SMAD4–CDH1 signaling. |
| Hee-Jung Choi et al. | BMB Reports | TGF-β1 directly induces the adhesion of endometrial cells to mesothelial cells through the regulation of integrin αV, α6, β1, and β4 via the activation of the TGF-β1/TGF-βRI/Smad2 signaling pathway. |
| Xiang Lin et al. | ENDOCRINOLOGY | The hypoxic microenvironment stimulates ESCs to produce excessive TGF-β1 and activates the TGF-β1/Smad signaling pathway, thus enhancing integrin expression and |

| | | |
|---|---|---|
| | | the adhesion ability of ESCs. |
| Hee-Jin Choi et al. | Experimental & Molecular Medicine | The increased sialification effect of TGF-β1 on endometrial cells promotes the adhesion of the endometrium to the peritoneum and the occurrence of endometriosis. |
| Upendra Kumar Soni et al. | Biology of Reproduction | TGF-β1 potentiates the adhesion of ectopic endometrial cells/tissues in the peritoneal region by enhancing the integrin and FAK signaling axis, and also migration via cadherin-mediated EMT and RHOGTPase signaling cascades. |
| Tian Gao et al. | Reproductive Biology | Depletion of NRP2 restrains ectopic ESC migration, invasiveness and EMT. TGF-β signaling-mediates activation of SMAD2 transcriptionally upregulated NRP2 expression in ectopic ESCs. TGF-β treatment could rescue NRP2 silencing-induced suppressive impact on the behaviors of ectopic ESCs. |
| Juan Zhang et al. | Reproductive Biology and Endocrinology | Knockdown of VCAM-1 impedes TGF-β1-mediated proliferation, migration, and invasion of endometrial cells, thereby indicating that VCAM-1 may serve as a therapeutic target for endometriosis. |
| Anca Chelariu-Raicu et al. | Fertility and Sterility | The upregulation of syndecan-4 in the eutopic endometrium of endometriosis patients may facilitate the pathogenetic process by promoting invasive cell growth via Rac1, MMP3, and ATF-2. |
| Sakthivignesh Ponandai-Srinivasan et al. | Human Reproduction | Compared with other low-expression samples (Endo-lo samples), the sample subgroup with a higher gene expression profile (Endo-hi samples) shows an abnormal pro-invasive response when TGF-β signal is activated. The invasive behavior of Endo-hi samples can be reversed by reducing the inherent high levels of SDC1 or SDC4 in combination with the activation of TGF-β signaling. |
| Fengyu Wang et al. | American Journal of Physiology Cell Physiology | TRIM59 inhibits PPM1A through ubiquitination and activates TGF-β/Smad pathway to promote the invasion of ectopic endometrial stromal cells in endometriosis. |
| Hwi Gon Kim et al. | International Journal of Molecular Sciences | DEHP promotes the development of endometriosis by activating the TGF-β/Smad signalling pathway, which triggers endometrial cell proliferation, migration, stemness (stem cell properties) and epithelial-mesenchymal transition (EMT). |

| | | |
|---|---|---|
| Yi Wen et al. | Cell Journal | β-sitosterol can inhibit the proliferation of endometrial cells and relieve endometriosis by inhibiting TGF-β-induced phosphorylation of Smads through regulation of Smad7. |
| Li Bing Shi et al. | Biology of Reproduction | Endometriotic cells of endometriomas synthesize TGF-beta1 leading to fibrosis and adhesion to ovarian tissues, and TGF-beta1/Smad signaling pathway is involved in this pathological process. |
| Xinliu Zeng et al. | Cellular Physiology and Biochemistry | The level of phosphorylated-NR4A1 is higher in ovarian endometriotic tissue than in normal endometrium, and long-term TGF-β1 stimulation phosphorylates NR4A1 in an AKT-dependent manner and then promotes the expression of fibrotic markers. Inhibition of NR4A1 in stromal cells increases the TGF-β1-dependent elevated expression of fibrotic markers, and loss of NR4A1 stimulated fibrogenesis in mice with endometriosis. Cytosporone B (Csn-B), an NR4A1 agonist, effectively decreases the TGF-β1-dependent elevated expression of fibrotic markers in vitro and significantly inhibited fibrogenesis in vivo. |
| Qi Zhang et al. | Molecular and Cellular Endocrinology | Activated platelets, through the release of TGF-β1, activate the TGF-β/Smad signaling pathway, promoting EMT and FMT in endometriosis, resulting in increased cell contractility, collagen production, and ultimately fibrosis. |
| Agnes N. Mwaura et al. | Biology | The canonical TGF-β pathway involving TGF-β/ALK-5/SMAD3 signaling is required in TGF-β-mediated reduction in the shedding of BG in endometriotic cells. Inactivation of SMAD3 but not of SMAD2 in endometriotic cells followed by treatment with TGF-β1/2 increases BG shedding, suggesting non-redundant SMAD3-dependent regulation of BG shedding. |
| Yue Xia et al. | American Journal of Translational Research | Neferine inhibits the progression of endometriosis both in vitro and in vivo. Its mechanism of action may involve the regulation of the TGF-β/ERK signaling pathway, leading to the inhibition of fibrosis in endometriosis. |
| Ayako Muraoka et al. | Science Translational Medicine | Fusobacterium infection promotes the development of endometriosis by activating the TGF-β signalling pathway, inducing the transformation of endometrial fibroblasts to |

| | | |
|---|---|---|
| | | TAGLN-positive myofibroblasts, and enhancing cell proliferation, adhesion and migration. |
| Hui-Li Yang et al. | Society for Reproduction and Fertility | The interaction between macrophages and ESCs downregulates cytotoxicity of NK cells possibly by stimulating the secretion of IL-10 and TGF-β, and may further trigger the immune escape of ectopic fragments and promote the occurrence and the development of EMS. |
| Kenji Ogawa et al. | Reproductive Sciences | TGF-β1 produced by endometriotic cells may protect against oxidative injury through the upregulation of macrophage-derived HO-1. The cross-talk between endometriotic cells and macrophages may contribute to the progression and pathogenesis of endometriosis. |
| Sixue Wang et al. | Archives of Gynecology and Obstetrics | The presence of miR-141, EMT, and TGF-β1/SMAD2 signalling markers are detected in eutopic and ectopic endometria of endometriosis. TGF-β1 induces EMT in Ishikawa (ISK) cells by activating the SMAD2 signalling pathway, whereas miR-141 inhibits the TGF-β1-induced EMT, proliferation and invasion abilities of these cells. |
| Qing Huan et al. | Journal of Obstetrics and Gynaecology Research | AFAP1-AS1 silencing can inhibit cell proliferation and promote apoptosis by regulating STAT3/TGF-β/Smad signaling pathway via targeting miR-424-5p in ESCs. AFAP1-AS1 may be a potential therapeutic target of controlling the progression of endometriosis. |
| Lin Sun et al. | Bioengineered | The circPIP5K1A accelerates EMS progression in vitro by activating the TGF-β signaling pathway via the miR-153-3p/TMSB4X axis, providing a potential clinical target for EMS treatment. |
| Na Li et al. | Reproductive Biology | The miR-143-3p activates TGF-β signaling by targeting VASH1 to facilitate migration and invasion of ESCs. |
| Jing Li et al. | Human Reproduction | Ecto-MSCs enhances the fibrotic behavior of stromal cells in ovarian endometrioma through the Wnt/β-catenin pathway by paracrine production of transforming growth factor-β1 (TGF-β1) and Wnt1. |

| | | |
|---|---|---|
| Ying Feng et al. | Journal of Obstetrics and Gynaecology Research | TGF-β1 secreted by ecto-MSCs facilitates fibrogenesis in EMs through SMAD3/DNMT3A-mediated RASAL1 inhibition. |
| Yuri Kadota et al. | Endocrine Journal | Activin A activates the SMAD signaling pathway and promotes the development of endometriotic lesions, thus identifying SMAD7 as a potential therapeutic target for endometriosis. |
| Chelsea J. Stoikos et al. | Human Reproduction | Decreased trophoblast CAM production and adhesion could be caused by dysregulated local activin A levels and may contribute to implantation failure. This could explain, in part, the infertility observed in women with EMS. |
| Osamu Yoshino et al. | Fertility and Sterility | Activin-A, which is induced by IL-1β or TNF-α, might promote endometriosis by stimulating IL-6 and PAR-2 mRNA expression and increasing the proliferation of EoSC. |
| Luk Rombauts et al. | The Australian and New Zealand Journal of Obstetrics and Gynaecology | The expression of inhibin/activin subunits in eutopic endometrium is altered in women with endometriosis, leading to higher levels of activin-A secretion by both glandular cells and stromal cells. |
| Ana Luiza L Rocha et al. | Reproductive Sciences | Activin A regulates the expression and secretion of IL-8 and VEGF in cultured HESC, and this mechanism appears to be disrupted in eutopic endometrial cells from women affected by endometriosis. |
| Juan Zheng et al. | BioMed Research International | Activin A promotes the secretion of estradiol from ESCs by increasing the expression of P450arom via the ALK4-Smad pathway. These findings indicate the ALK4-Smad pathway may promote ectopic lesion survival and development. |
| Juan Qu et al. | Reproductive Sciences | Activin A can stimulate aromatase P450 (P450arom) expression in eutopic endometrial stromal cells (ESCs) of endometriosis by activin type I receptor-Smad pathway. |
| Zhenzhen Zhang et al. | Cell Communication and Signaling | Activin A promotes myofibroblast differentiation of endometrial mesenchymal stem cells via STAT3-dependent Smad/CTGF pathway. |
| Zian Liao et al. | Nature Communications | The dysfunction of BMP/SMAD signaling in the endometrium of patients with endometriosis explains the deindividuation defects of these individuals and the |

| | | |
|---|---|---|
| | | subsequent pregnancy complications. |
| Farfaras Athanasios et al. | Archives of Gynecology and Obstetrics | Ectopic endometrium shows intense cytoplastic immunoreactivity to BMP-6 in both epithelium and stroma. BMP-6 expression is highly associated with strong expression of ERa. |
| Jianjuan Li et al. | Frontiers in Bioscience-Landmark | PKM2/HIF-1α-axis-dependent glycolysis participates in the pathogenesis of EP combined with endometriosis by mediating TGF-β1 signaling. |
| Tao Wang et al. | Molecular and Cellular Endocrinology | TGFβ1 inhibits the proliferation of CESCs and HESCs while significantly promoting the proliferation of EESCs. COX-2 modulates the effects of TGFβ1 on endometrial stromal cells by altering the balance between the Smad3 and ERK1/2 signaling pathways, thereby converting TGFβ1 from a growth inhibitor to a proliferation stimulator. |
